# High aspect ratio 10-nm-scale nanoaperture arrays with template-guided metal dewetting

**DOI:** 10.1038/srep09654

**Published:** 2015-04-10

**Authors:** Ying Min Wang, Liangxing Lu, Bharathi Madurai Srinivasan, Mohamed Asbahi, Yong Wei Zhang, Joel K. W. Yang

**Affiliations:** 1 Institute of Materials and Research Engineering, A*STAR, Singapore 117602, Singapore; 2 Institute of High Performance Computing, A*STAR, Singapore 138632, Singapore; 3 Singapore University of Technology and Design, Singapore 138682, Singapore

## Abstract

We introduce an approach to fabricate ordered arrays of 10-nm-scale silica-filled apertures in a metal film without etching or liftoff. Using low temperature (<400°C) thermal dewetting of metal films guided by nano-patterned templates, apertures with aspect ratios up to 5:1 are demonstrated. Apertures form spontaneously during the thermal process without need for further processing. Although the phenomenon of dewetting has been well studied, this is the first demonstration of its use in the fabrication of nanoapertures in a spatially controllable manner. In particular, the achievement of 10-nm length-scale patterning at high aspect ratio with thermal dewetting is unprecedented. By varying the nanotemplate design, we show its strong influence over the positions and sizes of the nanoapertures. In addition, we construct a three-dimensional phase field model of metal dewetting on nano-patterned substrates. The simulation data obtained closely corroborates our experimental results and reveals new insights to template dewetting at the nanoscale. Taken together, this fabrication method and simulation model form a complete toolbox for 10-nm-scale patterning using template-guided dewetting that could be extended to a wide range of material systems and geometries.

The patterning of 10-nm-scale nanoapertures at high aspect ratio is potentially of great interest to biological sensing^1^,^2^,^3^,^4^, x-ray diffraction^5^,^6^,^7^, and the study of plasmonics^8^,^9^. However, existing methods to controllably fabricate nanoaperture arrays in metal films are inadequate in terms of achievable resolution and aspect ratio, scalability and cost. Amongst the established nanofabrication techniques, helium ion-beam milling and metal liftoff techniques have been demonstrated to achieve 10-nm scale patterning without additional modifications.

Helium ion-beam milling, in particular, has been shown to achieve sub-10-nm resolution at high aspect ratios^9^, albeit at a low milling rate and at considerably high costs. These disadvantages, along with the inherently low throughput of serial milling techniques, limit the practical use of helium ion beam milling to device prototyping. Metal liftoff has recently been applied to achieve 10-nm-scale trenches in gold films at an aspect ratio of ~3:1^10^. However, the attempt to obtain nanoapertures at the same scale fails due to significant sidewall deposition that impedes liftoff^10^. Furthermore, as a rule of thumb, the resist thickness needs to be three times greater than the desired metal thickness in order to ensure reasonable yield. This requirement poses an inherent limitation to the aspect ratio of apertures achievable with metal liftoff because of the challenges associated with high aspect ratio lithography of nanotemplates, e.g. reduced lithographic resolution due to forward scattering of electrons, and nanostructure collapse. Although novel techniques utilizing atomic layer deposition have recently been reported to achieve extremely small nanogaps, these techniques are not yet amenable to the patterning of circular nanoapertures^11^,^12^. There is, thus, a pertinent need for a simple, reliable, and cost-effective fabrication method that can potentially enable scalable production of nanopertures.

The dewetting behavior of thin films has been extensively investigated both experimentally and theoretically, with interests in its fundamental physics and its promise as an economical approach to obtain nanostructured surfaces and nanodevices^13^,^14^,^15^,^16^,^17^,^18^,^19^,^20^. Deposited metallic films are often thermodynamically out of equilibrium due to their limited wettability on substrates, and local substrate defects and topography. When the sample is heated up, the metallic film dewets, evolving into more thermodynamically stable configurations by minimizing free energy, e.g. by rupturing and clustering to form a random arrangement of nanostructures with a large spread in sizes^14^,^16^. To fulfill the potential of dewetting as a nanofabrication method, deterministic control over the sizes and positions of the nanostructures is required. Recently, ordered arrays of 100-nm-scale nanoparticles have been demonstrated through dewetting of pre-patterned metal films^21^,^22^, and metal films deposited on topographically patterned substrates^23^,^24^. Although the spontaneous formation of nanoscale holes over the course of dewetting has been shown^13^,^14^,^15^,^18^,^20^, position and size control remains a challenge. Moreover, these holes are often in metastable states that quickly evolve into more complex perforated structures, and eventually cluster as the system is driven further towards equilibrium.

In this article, we present a novel method that enables the fabrication of nano*aperture* arrays obtained through dewetting of metals on nanopatterned templates. We report the ability to obtain 10-nm scale apertures with aspect ratios up to 5:1 in a controllable and spatially defined manner. Furthermore, we detail a careful and comprehensive study that explores the parameter space for our approach and further expounds on the mechanisms of dewetting of metal on substrates. Our experimental findings are closely corroborated by a three-dimensional numerical model that provides insights to the mechanisms of the physical processes of our method.

## Results and Discussion

**Figure 1:**
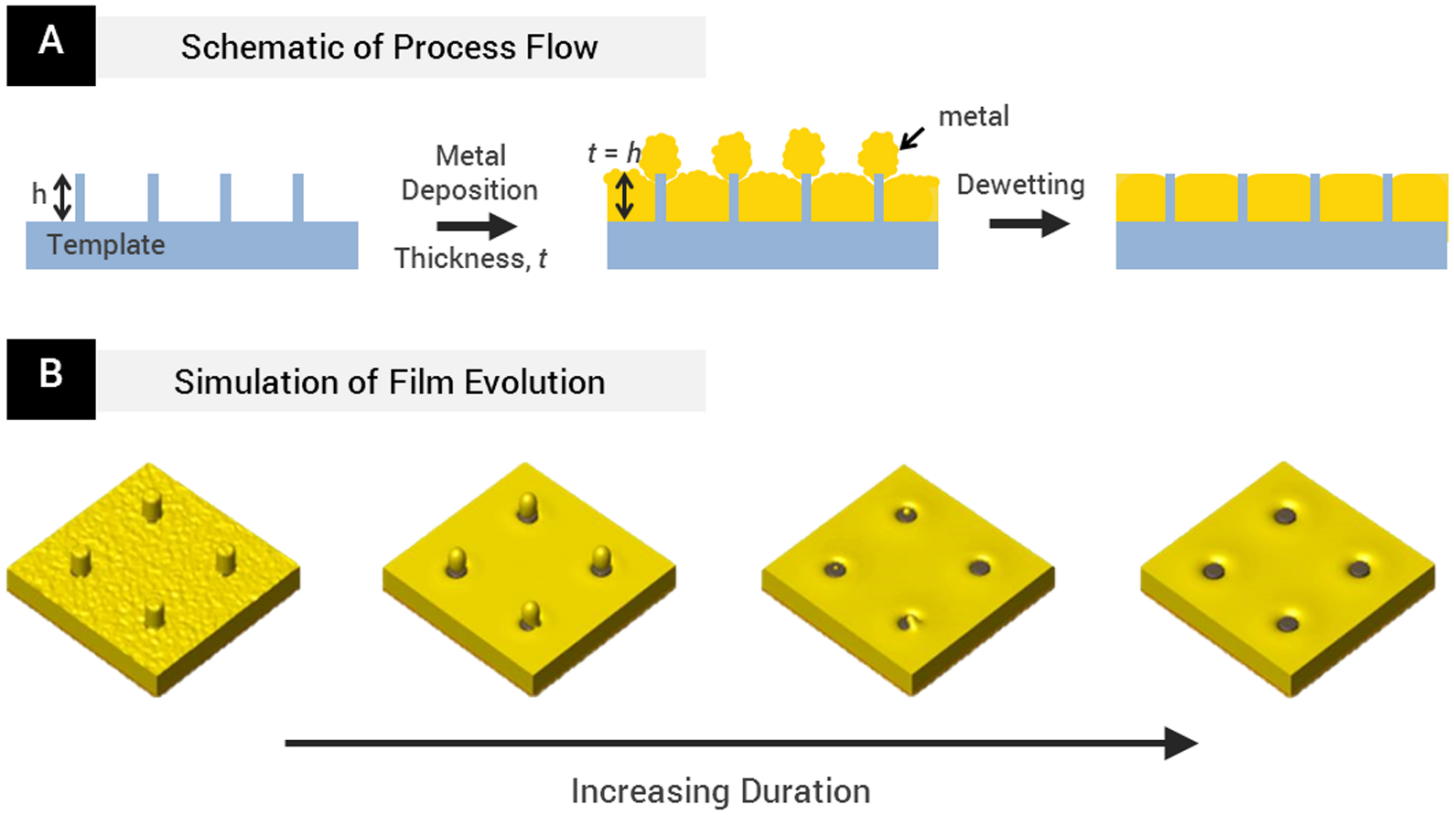
(A) Schematic illustrating the fabrication process. A template consisting of nanopillars with height, *h*, is used. Next, metal of thickness, *t*, equal to *h*, is deposited via electron beam deposition. Finally, solid-state dewetting is induced at low temperature (<400°C). As a result of capillary-assisted diffusion, the metal caps on top of the nanopillars dewet away from the nanopillars into the surrounding metal film, revealing nanoapertures. (B) Simulated time-lapse view of the dewetting process obtained using a three-dimensional phase field model. The metal caps atop the nanopillars ball up and form joints with the surrounding film. With increasing duration, the metal caps are drawn away from the tops of the nanopillars into the surrounding film.

Figure 1A shows a schematic of our method. Here, we use nanopillars as templates, onto which metal is deposited. Unlike in liftoff processes, the thickness of the metal deposited here is comparable to the height of the nanopillars. The locations and dimensions of the transparent nanopillars register the final positions of the nanoapertures and determine their diameters respectively. With low temperature thermal annealing, the metal caps dewet off the top of the pillars and then coalesce with the surrounding metal film, revealing the top surfaces of the optically clear nanopillars. To validate the concept of the method and to obtain further insights, we construct a three-dimensional phase field model, which elucidates the evolution of the system by solving the Cahn-Hilliard equation via the Fourier-Spectral method at regular time steps^25^. Details of the simulation model are presented in Supplementary Information.

Figure 1B shows the simulated time evolution of the deposited film. In the simulation, surface roughness is introduced to the gold film at its initial state to match the roughness of the as-deposited film. The simulation results show that the edges of the gold caps on the nanopillars initially ball up to minimize surface energy, and the film roughness rapidly decreases as the total free energy in the system is minimized. When the nanopillar templates are designed in such a way that it is energetically unfavorable for gold caps to remain on the top of the nanopillars (see our analysis and discussion later), the gold caps merge into the surrounding film through the thermally accelerated diffusion of the gold atoms. Eventually, the gold caps diffuse completely into the surrounding metal film, revealing the nanoapertures. (See also Supplementary Video M1 of simulated film evolution).

**Figure 2:**
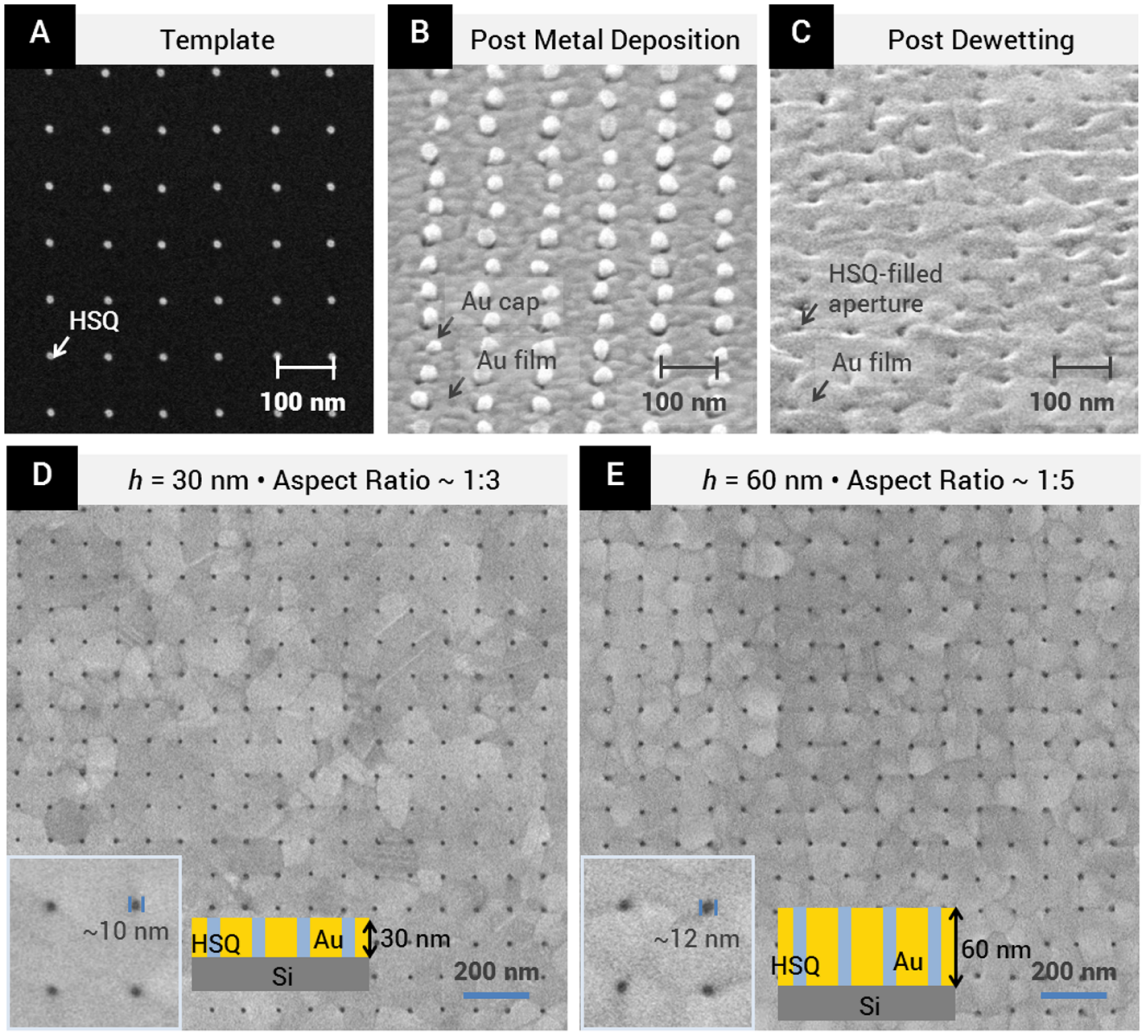
Experimental results showing the template-guided metal dewetting process. (A) SEM image of HSQ defined nanopillars on bare silicon. Periodicity of nanopillars = 100 nm. (B) Tilted SEM image of sample after metal deposition. (C) Tilted SEM image of nano-apertures obtained after thermally induced dewetting. The grain sizes increased significantly after thermal treatment. (D) SEM micrograph of nanoaperture array in 30 nm thick film obtained over a ~1.5 μm by 1.5 μm area. Scale bar: 200 nm. (E) SEM micrograph of nanoaperture array in 60 nm thick film obtained over a ~1.5 μm by 1.5 μm area. (D & E - insets) High magnification SEM images showing a close-up of the nanoapertures in D and E respectively.

To experimentally demonstrate this process, we fabricated nanopillar templates on silicon substrates with native oxide intact. These 10-nm-scale nanopillars were defined using electron beam lithography (EBL) crosslinking of hydrogen silsesquioxane (HSQ), a high contrast negative-tone resist^26^,^27^,^28^. Cross-linked HSQ resembles amorphous SiO_x_ and is optically clear. Figure 2A shows a fabricated template consisting of HSQ nanopillars ~ 10 nm in diameter, 30 nm in height, and at a periodicity of 100 nm. Thirty nanometers of gold was deposited on the template via electron-beam evaporation. The as-deposited gold film was continuous except at the locations of the nanopillars, on top of which gold caps were formed. As seen from the SEM image taken at a tilted angle (Figure 2B), these metal caps were mostly physically disconnected from the metal film deposited on the substrate. Next, metal dewetting was induced via thermal processing at 400°C in a rapid thermal processor for 5 minutes. Details of the fabrication process are provided in the *Methods* section.

As predicted by our simulations, the dewetting process resulted in the diffusion of gold caps off the top of the nanopillars and into the metal film surrounding the nanopillars, revealing HSQ-filled nanoapertures as shown in the tilt-view SEM image in Figure 2C. Figure 2D shows an SEM image of a nanoaperture array obtained over a ~ 1.5 by 1.5 μm^2^ area. The achievable yield was high, with few missing apertures that may be attributed to collapsed HSQ nanopillars. We found that the mean diameters of the nanoapertures obtained via dewetting were ~10 nm, closely matching that of the HSQ nanopillars. (See Supplementary Information Figure S1 for nanopillar and nanoaperture diameter distributions.) We note that these reported measurements are conservative because the varying gold crystal orientations resulted in different local signal levels on the SEM micrograph, potentially broadening the spread of diameter measurements. In addition, we found that this process is insensitive to differences between the thickness of metal deposited and the pillar height (<±5 nm) as shown analytically and experimentally in the Supplementary Information.

The fabrication of high aspect ratio nanoapertures is of particular interest for mechanically robust and optically opaque metal films. A significant advantage of our method is that the nanopillars need only to be as tall as the desired metal film thickness. This substantially reduces the constraint on template aspect ratio, availing our method for practical fabrication of high resolution and high aspect ratio nanoapertures. To demonstrate this advantage, we used templates consisting of nanopillars with height of 60 nm and diameters of ~12 nm, corresponding to an aspect ratio of ~5:1. Sixty nanometers of gold was deposited, after which dewetting was thermally induced at 400°C for 10 minutes. Figure 2E shows a nanoaperture array obtained at a periodicity of 100 nm, in a 60 nm thick film, covering a ~ 1.5 by 1.5 μm^2^ area. We note that the resolution and aspect ratio obtained here does not represent the ultimate limit of our method. The extent of our demonstration is currently constrained by the lithographic resolution and the mechanical robustness of HSQ nanopillars, which tend to collapse at high aspect ratios^10^,^29^,^30^. It is reasonable, however, to expect that the aspect ratio of the nanopillar templates can be increased significantly with the use of a critical point dryer or with templates materials with greater mechanical strength.

**Figure 3:**
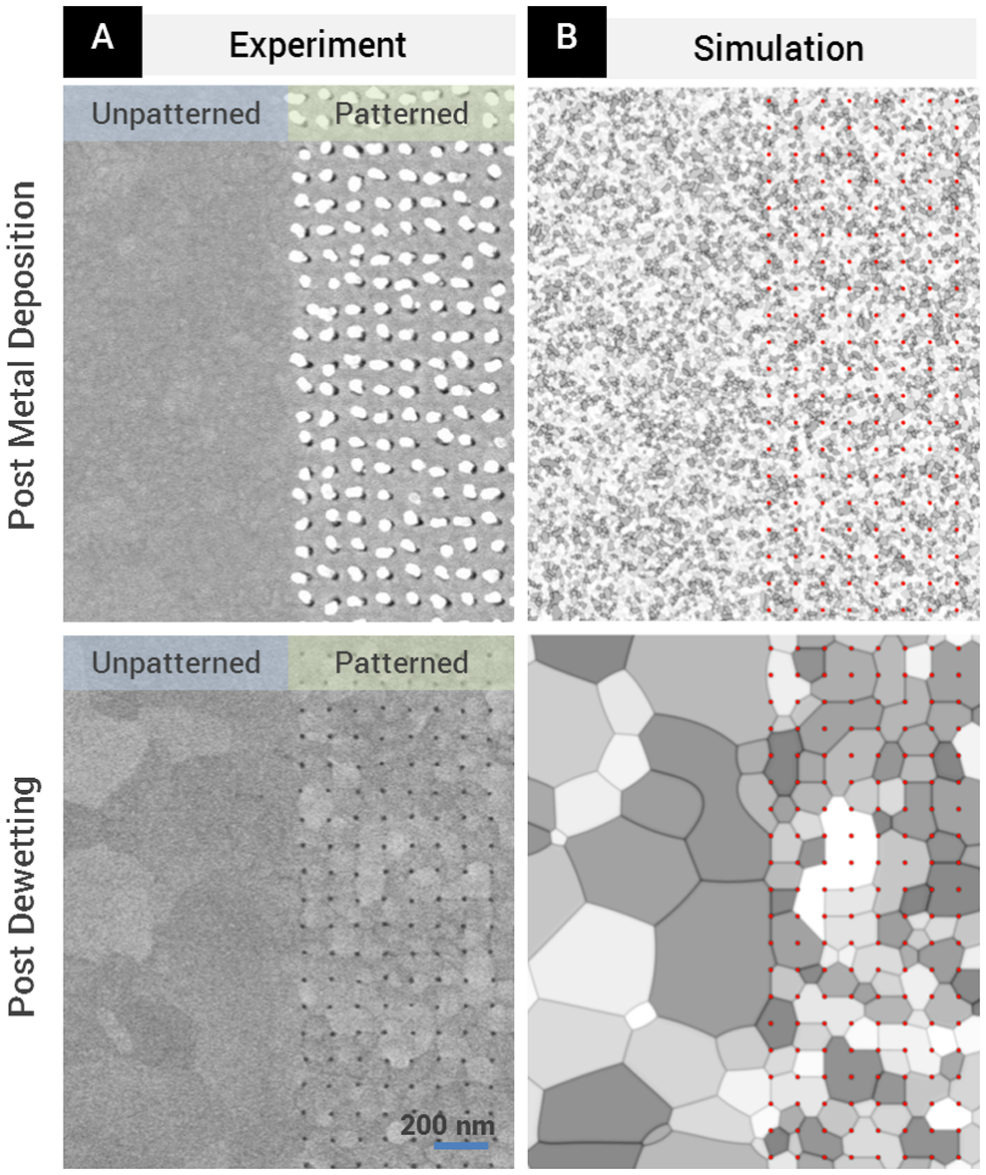
Patterned area versus an unpatterned area before and after dewetting. (A) SEM micrograph of samples with gold film thicknesses of 60 nm, and nanoaperture periodicity of 100 nm. Thermal annealing induces recrystallization of the metal, with noticeable grain-boundary pinning yielding smaller grain sizes in the patterned versus the unpatterned areas. (B) In the simulation results, red dots indicate positions of the nanopillars (with gold caps) and nanoapertures before and after dewetting respectively. The simulation results agree with the experimental findings, showing the overall increase in grain sizes after dewetting, and grain-boundary pinning in the patterned areas resulting in smaller grain sizes as compared to unpatterned areas.

An inherent increase in metal film crystallinity is another important advantage of our method. The increase in crystallinity after dewetting was clearly observed in our nanoaperture arrays (comparing Figures 2B and 2C, and from Figure 3A), as was also predicted in our simulations where the initial roughness of the metal film was shown to quickly smoothen out over the course of phase field evolution (Figure 1B and Figure 3B). The experimental data and simulations results shown in Figure 3 further confirmed that the nanoapertures formed only in the patterned area and that no spontaneous dewetting occurred in the unpatterned area. Pinning of grain boundaries by the nanopillars was also observed, as can be seen in the smaller grain sizes in the patterned regions, as compared to the unpatterned regions (Figure 3). In addition to the significant increase in crystallinity, metal adhesion layers are not a prerequisite because our method does not involve physical agitation or immersion in solvents as required in lift-off techniques. The ability to fabricate highly crystalline gold nanostructures without the need for an adhesion layer highly desirable in plasmonic applications, where grain boundaries, surface roughness and adhesion layers are known to significantly contribute to plasmon damping^31^,^32^,^33^,^34^. Thus, our fabrication method can potentially enable more sensitive devices with higher Q-factors^31^,^32^.

**Figure 4:**
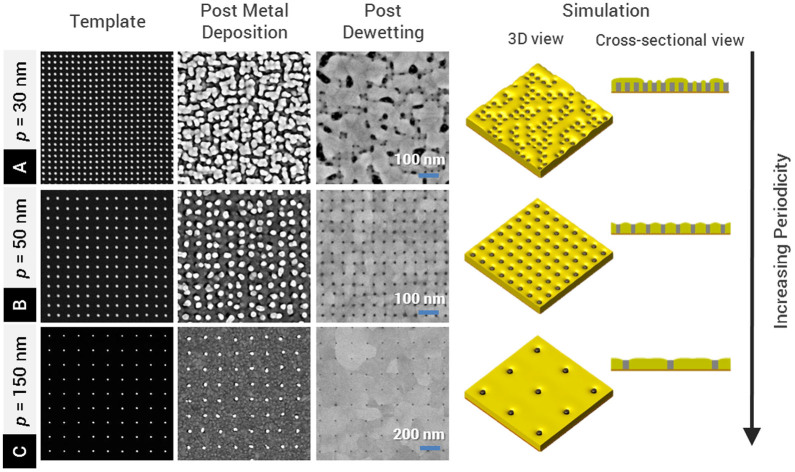
Effect of nanopillar periodicity on fabrication outcome. SEM micrographs of the templates, the samples after gold deposition and the samples after dewetting are shown for nanopillar periodicities (A) *p* = 30 nm, (B) *p* = 50 nm, and (C) *p* = 150 nm. Phase field simulation results were also shown for all three periodicities. At *p* = 30 nm, after gold deposition, the neighboring gold caps were joined due to the small spacing. Thermal treatment did not yield nanoaperture array at this periodicity. At *p* = 50 nm and larger, the gold caps dewet off nanopillars, resulting in clear nanoapertures. We note that the positions of the gold caps after gold deposition appeared to deviate from the regularity of the nanopillars arrays. These positional jitters appeared to be partially removed with thermal treatment, resulting in nanoapertures with more regular periodicities.

Because dewetting is a physical process driven by the reduction in total free energy of the system, we expect the nanopatterned template geometry (e.g. nanopillar periodicity and diameter) to play an important role in the fabrication outcome. Here, we investigate this dependence. Experimentally, we varied the periodicity of the nanopillar templates from 30 nm to 150 nm. Figure 4 shows representative scanning electron micrographs of HSQ nanopillar templates of increasing periodicity, and samples post metal deposition and post dewetting. Corresponding simulation results are also presented. At a periodicity of 30 nm, we found that the as-deposited metal caps coalesced (Figure 4A), and did not yield nanoaperture arrays after thermal processing. In addition, ruptures in the film were observed at various locations. However, at periodicities larger than 30 nm (Figure 4B and C), nanoapertures were successfully obtained.

The reason for the lower limit in periodicity to yield nanoapertures can be understood by considering the energetics of the system over the process of dewetting. The interfacial energy per unit volume between gold and SiO_x_ (HSQ and native oxide layer on silicon substrate) increases as the periodicity of the HSQ nanopillars decreases. In order to minimize the interfacial energy at small periodicities, the deposited metal is expected to dewet from the SiO_x_ interface to undesirably result in the Cassie–Baxter (CB) state where the metal forms a continuous film on top of the nanopillars^35^, leaving bare nanopillar sidewalls (Figure S2A). As derived in the Supplementary Information, the critical periodicity at which the CB state occurs can be analytically determined to be ~50 nm, with 30 nm nanopillar height and 10 nm nanopillar diameter. This agrees well with the experimental observations (Figure 4a). In the simulations shown in Figure 4a, for *p* = 30 nm, a metastable Wenzel (wetting) state^36^ was observed within time steps corresponding to the experimental time frame but evolves to the experimentally observed CB state, when driven closer to equilibrium (see details in the Supplementary Information Figure S2B). This subtle difference can be attributed to a sensitive dependence of the final structures on the initial geometries of the metal before annealing.

**Figure 5:**
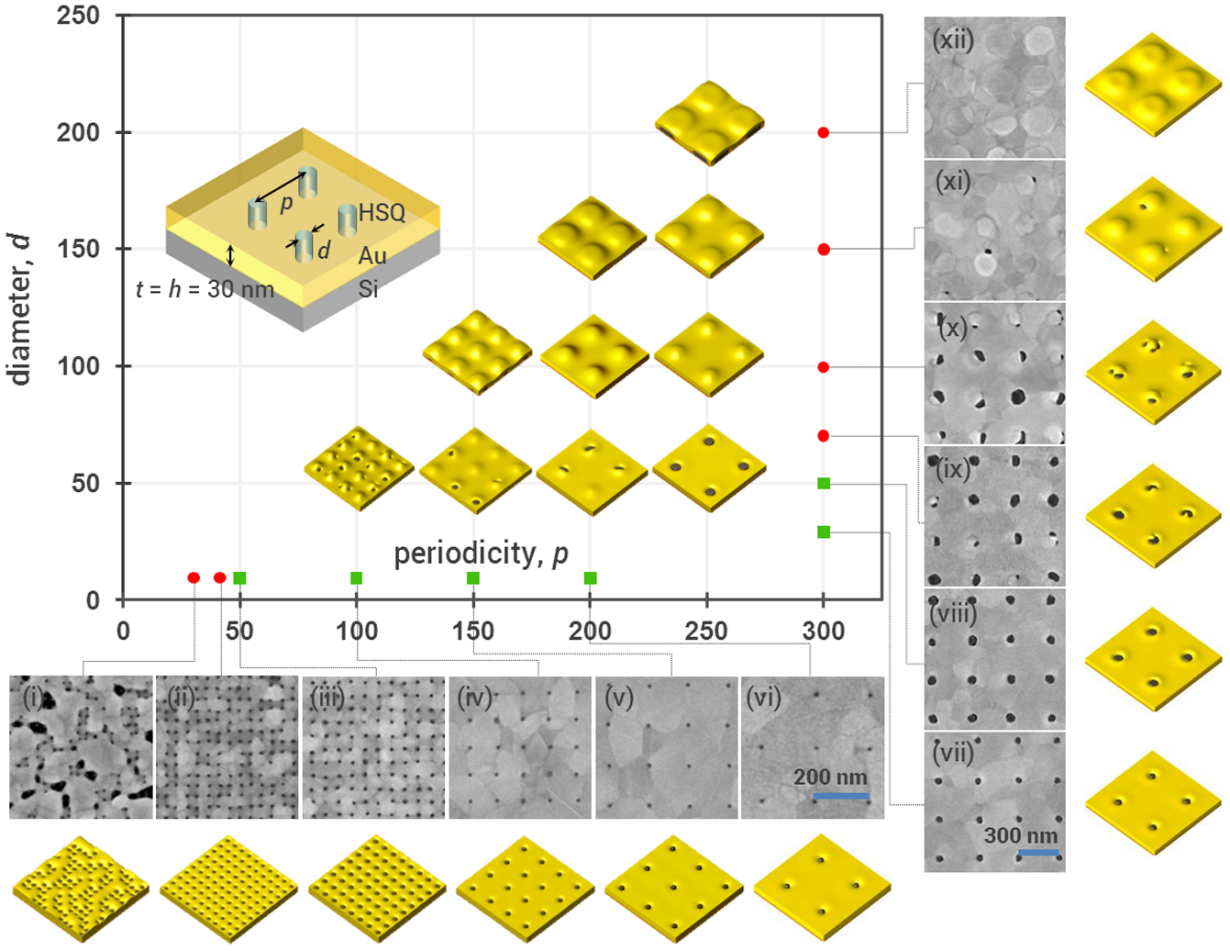
Parameter variations with simulations and experiments for *t* = *h* = 30 nm. Experimental results were obtained for (i–vi) *d* ~ 10 nm, *p* = 30 nm, 40 nm, 50 nm, 100 nm, 150 nm, and 200 nm respectively; (vii–xii) *p* = 300 nm, *d* = 30 nm, 50 nm, 70 nm, 100 nm, 150 nm, and 200 nm respectively. Simulation results were obtained for the entire parameter space, and displayed here over an area of 400 nm by 400 nm. Green squares indicate experimental parameters with which nanoaperture arrays were achieved. Red dots indicate parameters with which dewetting fails to produce aperture arrays. Corresponding results for *t* = *h* = 60 nm are shown in supplementary figure S4.

The close agreement between the experimental and simulation results obtained indicates that the model presented here successfully captures the physical processes governing the fabrication method. In fact, the viability of this method with various nanopillar configurations (diameter, height, and periodicity) can be similarly investigated. Exploiting this advantage, we performed a parameter sweep to evaluate the feasibility of our method over a range of periodicities and diameters. Figure 5 shows the experimental results obtained for apertures 30 nm deep (*h* = 30 nm), with diameters *∼* 10 nm, and periodicities ranging from 30–200 nm; and larger apertures with diameters ranging from 30 to 100 nm, and a fixed periodicity of 300 nm. Simulation results are shown over the entire parameter space. Thermal treatment time and the corresponding number of simulation time steps were kept constant across all parameters investigated.

We found that the fabrication method is robust and versatile, allowing for the fabrication of apertures 10 nm to 50 nm in diameter, with periodicities more than approximately four times the aperture diameter. In the regime where the diameters of the nanopillars were>1/4 that of the periodicities, the gold caps on top of the nanopillars did not completely dewet, which can be understood in terms of energetics as discussed. Remarkably, in the transition region where the diameters of the nanopillars were ~1/4 of the nanopillar periodicities, simulations predict that increased thermal treatment time would eventually yield nanoapertures (Supplementary Information Figure S3), suggesting the importance of optimizing thermal treatment time. A similar study for the case of *h* = 60 nm is presented and discussed in Supplementary Information Figures S4 and S5.

**Figure 6:**
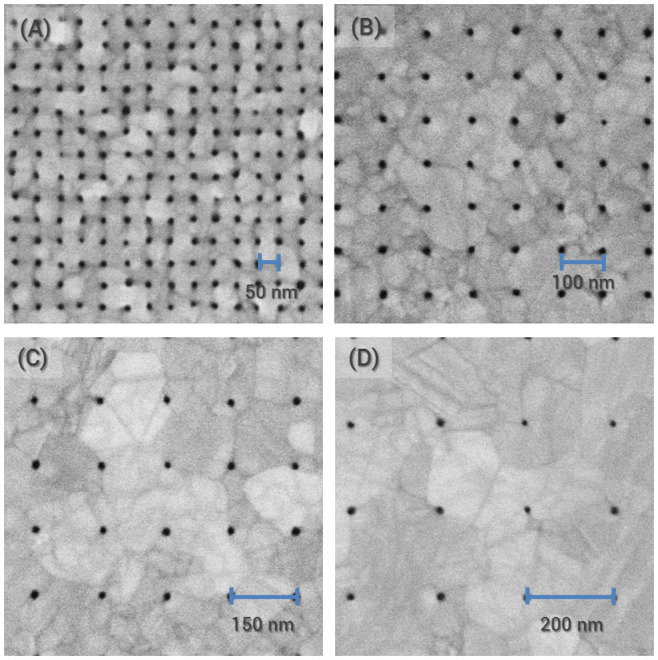
Nanoapertures obtained by annealing on a hotplate at 250°C for 15 minutes. Scanning electron micrographs showing 10 nm-scale apertures at periodicities of (A) 50 nm, (B) 100 nm, (C) 150 nm, and (D) 200 nm.

Although we have demonstrated this fabrication method using a specific materials system – native oxide on Si substrate, HSQ, and gold, we expect similar results with other combinations of materials as long as the film, metal or otherwise, wets the template in a similar way. (See Supplementary Information Figure S6 for SEM micrographs of nanoapertures obtained with templates consisting of HSQ nanopillars on thermally grown silicon oxide and PECVD silicon nitride substrates.) For materials with lower diffusivity, the dewetting temperature and time can be increased to obtain similar results. The simulation model presented here provides a viable means to investigate the feasibility and to aid in the optimization of our fabrication method with a variety of material systems, and initial metal film and nanotemplate configurations. In applications where vacant or via holes are desired, the nanotemplate material can be removed gently through wet or dry etching. In the case of our demonstration, the HSQ nanopillars can be removed using hydrofluoric acid and via holes can be obtained by etching through the silicon substrate. However, unlike conventional processes that require the removal of template materials in order to obtain apertures, the dewetting process allows for the template materials to remain inside the metal holes. We note that a recent method to obtain glass-filled nanoapertures has been demonstrated, requiring the mechanical cleaving of gold-capped template structures to obtain apertures^37^. In contrast, our demonstration does not require further processing after thermal treatment. Finally, our process is amenable to low temperature processing, avoiding high temperature induced damage to the template or undesirable alloying between the metal and the template materials. We have, in fact, shown successful fabrication by heating the sample on a standard laboratory hotplate at 250°C for 15 minutes (Figure 6).

## Conclusion

We introduced a readily implementable approach to achieve10-nm-scale nanoaperture arrays in metal films with high aspect ratios utilizing template-guided low temperature solid-state dewetting. With gold and HSQ nanopillars on silicon templates as a model system, we demonstrated the applicability of this technique for the fabrication of 10–50 nm nanoaperture arrays with aspect ratios up to 5:1, length scales that had previously been prohibitively difficult to achieve. We expect that this approach would yield sub-10-nm nanoapertures with even higher aspect ratios, limited only by the resolution of the appropriately fabricated templates. Three-dimensional phase field simulations provide insights into the mechanism of the dewetting process and closely match experimental findings. The simulations accurately predict the success of the fabrication method with various template configurations, and inform on possible means for optimization. With templates that can potentially be defined with nanoimprint lithography and the compatibility of the process with low temperature processing, our fabrication method can potentially enable scalable and cost-effective production of nanopore-based biological and chemical sensing devices; and find applications in emerging nanoaperture-based phenomena and technologies such as in plasmonics, nanofiltration, and X-ray diffraction and EUV optics. Beyond the fabrication of nanoapertures, the experimental and simulations methods developed here will guide the design and fabrication of nanostructures and devices through template-guided dewetting in a wide variety of material systems and geometries.

## Methods

### Electron beam lithography

HSQ (Product number: XR-1541-002 (2%)/XR-1541-006 (6%), Dow Corning, Michigan, USA) was spun coated onto a silicon substrate to thicknesses of 30 nm and 60 nm. To obtain a resist thickness of 30 nm, 2% HSQ was spun-coated at 4000 rpm. To obtain a resist thickness of 60 nm, 6% HSQ was diluted in MIBK in 1:1 vol:vol ratio and spun-coated at 4000 rpm. The resist thickness is determined by ellipsometry and further confirmed by SEM imaging of fallen HSQ pillars (Figure S7). In order to achieve high resolution patterning, the resist was not pre-baked to avoid thermal crosslinking. Electron beam lithography (EBL) was performed using an Elionix ELS-7000 system with an accelerating voltage of 100 kV and a beam current of 500 pA. The exposure field size was 75 μm × 75 μm with a step size of 0.3125 nm. To obtain 10 nm scale structures, dot exposures with a typical dwell time of 70–100 μs were used. Structures up to 200 nm in diameter are also fabricated to investigate the upper size limit of the method. Development of exposed HSQ is done in a salty developer (1% NaOH + 4% NaCl wt/wt in DI water) at room temperature for 1 minute. To stop the development, the sample was immersed in DI water for 1 minute. Then, the sample was immediately immersed in IPA before blow-drying with a steady stream of nitrogen gas.

### Metal deposition

Gold, thickness comparable to the height of the HSQ nanostructures (30 nm and 60 nm), was deposited with an electron-beam evaporator (Explorer Coating System, Denton Vacuum, New Jersey, USA) at 0.5 Å/s. The chamber pressure during the process was ~5 × 10^−7^ torr. The sample holder was set to rotate at a rate of 12 rpm to ensure uniformity of the deposited metal film. No adhesion layer was used.

### Thermally induced dewetting

To induce thermal dewetting from the HSQ nanopatterns, the sample was heated up using a rapid thermal processing system (Jetfirst, Jipelec, Grenoble, France) with the following schedule: 120 sec ramp up from room temperature to 400°C, 5 minutes (30 nm thick gold) or 10 minute (60 nm thick gold) at 400°C, and 30 s ramp down to room temperature. We have also demonstrated dewetting by heating the sample on a hotplate at 250°C for 15 minutes. At the end of the thermal treatment, the sample was removed from the hotplate and immediately placed on a stainless steel surface to cool down.

### Scanning electron microscopy characterization

The samples, at each step, were characterized using scanning electron microscopy (Elionix Inc., Tokyo, Japan) at 5 or 10 kV with a working distance of 5 mm.

### Simulations

A three-dimensional phase field simulation model was developed. Briefly, the three-dimensional evolution of the phase fields is obtained by solving the Cahn-Hilliard equation using the Fourier-Spectral method. The simulations are performed on a workstation using a custom algorithm written in MATLAB (Mathworks, Massachusetts, USA). For periodicities of below 50 nm, simulation meshes of 32 divisions per period were used. For periodicities above 50 nm, simulation meshes of 64 divisions per period were used. The details of the model and the parameters used are provided in the Supplementary Information.

## Author Contributions

Y.M.W., M.A. and J.K.W.Y. designed the experiments. Y.M.W. performed the experiments. L.L., B.M.S. and Y.Z. designed the simulations. L.L. performed the simulations. Y.M.W., L.L., Y.Z. and J.K.W.Y. wrote the manuscript. All authors reviewed the manuscript.

## Supplementary Material

Supplementary Information Supplementary Information

Supplementary Information Supplementary Information
