# Supplementary material for: High aspect ratio 10-nm-scale nanoaperture arrays with template-guided metal dewetting
Source: Sci Rep. 2015 Apr 10;5:9654. doi: 10.1038/srep09654 (PMC4392361; doi:10.1038/srep09654)
Supplement: Supplementary Information [file srep09654-s1.pdf]

Supplementary Information

High aspect ratio 10-nm-scale nanoaperture arrays with template-guided metal dewetting

Ying Min Wang[1,‡], *Liangxing Lu*[2,‡], *Bharathi Madurai Srinivasan*[2], *Mohamed Asbahi*[1],

Yong Wei Zhang[2,*], *Joel K.W. Yang*[1,3,*]

[1] Institute of Materials and Research Engineering, A*STAR, Singapore 117602, Singapore

[2] Institute of High Performance Computing, A*STAR, Singapore 138632, Singapore

[3] Singapore University of Technology and Design, Singapore 138682, Singapore

‡These authors contributed equally.

[*] To whom correspondence should be addressed.

Corresponding emails: joel_yang@sutd.edu.sg & zhangyw@ihpc.a-star.edu.sg

Three-dimensional phase field simulations

We first describe the theory employed in our simulation model before delving into the details of the simulation algorithm.

Theory. In our phase field model, four conserved order parameters, $\varphi_1(\mathbf{r})$, $\varphi_2(\mathbf{r})$, $\varphi_3(\mathbf{r})$ and $\varphi_4(\mathbf{r})$, are employed to denote the phase distribution; specifically, $[\varphi_1,\varphi_2,\varphi_3,\varphi_4]=[1,0,0,0]$ denotes the vacuum phase, $[\varphi_1,\varphi_2,\varphi_3,\varphi_4]=[0,1,0,0]$ denotes the metal film phase, $[\varphi_1,\varphi_2,\varphi_3,\varphi_4]=[0,0,1,0]$ denotes the substrate phase, and $[\varphi_1,\varphi_2,\varphi_3,\varphi_4]=[0,0,0,1]$ denotes the nanopillars phase, with **r** being the position vector of a point, and constraint: $\sum_{i=1\sim 4}\varphi_i=1$ in the whole simulation region. To model the polycrystalline structure of the metal film shown in Figure 3, we also introduce another series of non-conserved order parameters η_i, with each η_i representing a random crystallographic orientation. In all other simulations, the polycrystalline nature of the metal film was not considered for simplicity. The coarse-grained Ginzburg-Landau free energy of the multiphase system, including bulk free energy, surface energies, interfacial energy and grain boundary energy (considered only in Figure 3), was modeled using the following free-energy function:

$$F^{tot}=\int\left[g_\varphi(\varphi_i)+A\varphi_2^2\cdot g_\eta(\eta_i)+B(1-\varphi_2)^2\sum_{i=1}^{m}\eta_i^2+f_{grad}\right]dV \qquad \text{(S1)}$$

where A and B are the system constants, g_φ and g_η are the bulk free energy of phases and grains, respectively. f_{grad} is the gradient free energy. The third term in Equation (S1) serves as a

pinning effect to the motion of grain boundaries, and is only considered in the simulations shown in Figure 3. Expressions of g_φ, g_η and f_{grad} are given below:

$$g_\varphi(\varphi_i) = \sum_{i<j}^{3}\left(\alpha_{ij}\varphi_i^2\varphi_j^2\right) \qquad \text{(S2)}$$

$$g_\eta(\eta_i) = \frac{1}{4}\sum_{i=1}^{m}(\eta_i^2 - 1)^2 + \frac{3}{4}\sum_{i<j}^{m-1}\eta_i^2\eta_j^2 - \frac{m-1}{4} \qquad \text{(S3)}$$

$$f_{grad} = -\sum_{i<j}^{3}\left(k_{ij}^{\varphi}\nabla\varphi_i\nabla\varphi_j\right) - \sum_{i<j}^{m-1}\left(k^{\eta}\nabla\eta_i\nabla\eta_j\right) \qquad \text{(S4)}$$

where α_{ij} are constants related to the height of double-well potential, k_{ij}^{φ} and k^{η} are the gradient energy coefficients for the phases and grains, respectively. According to the free energy terms given above, the surface/interface energy density can be expressed as:

$$\xi_{ij}^{\varphi} = \sqrt{2\alpha_{ij}k_{ij}^{\varphi}}\Big/3 \qquad \text{(S5)}$$

and grain boundary energy density can be expressed as:

$$\xi^{\eta} = \sqrt{2Ak^{\eta}}\Big/3 \qquad \text{(S6)}$$

To simplify the evolution equation, we assume that the phase field φ_3 and φ_4 describing the evolution of Si substrate and HSQ nanopilllars are static, i.e., they do not evolve with time. Since $\sum_{i=1\sim4}\varphi_i = 1$, we choose φ_2 as an independent phase field and its evolution follows the Cahn-Hilliard equation [1]:

$$\frac{\partial \varphi_2}{\partial \tau} = \nabla M \nabla \frac{\delta F^{tot}}{\delta \varphi_2} \qquad \text{(S7)}$$

and the kinetic evolution of grains is modeled by another non-conserved equation:

$$\frac{\partial \eta_i}{\partial \tau} = -L \frac{\delta F^{tot}}{\delta \eta_i} \qquad \text{(S8)}$$

where, M and L are the mobilities.

Numerical algorithm. Equation (S7) and (S8) are solved in their non-dimensional forms:

$$\frac{\partial \varphi_2}{\partial \tau^*} = \Delta^* \frac{\delta F^{tot*}}{\delta \varphi_2} \qquad \text{(S9)}$$

$$\frac{\partial \eta_i}{\partial \tau^*} = -L^* \frac{\delta F^{tot*}}{\delta \eta_i} \qquad \text{(S10)}$$

$$F^{tot*} = \int \left[g_\varphi^*(\varphi_i) + A^* \varphi_2^2 \cdot g_\eta(\eta_i) + B^*(1-\varphi_2)^2 \sum_{i=1}^{m} \eta_i^2 + f_{grad}^* \right] dV \qquad \text{(S11)}$$

$$g_\varphi^*(\varphi_i) = \sum_{i<j}^{3} \left(\alpha_{ij}^* \varphi_i^2 \varphi_j^2 \right) \qquad \text{(S12)}$$

$$f_{grad} = -\sum_{i<j}^{3} \left(k_{ij}^{\varphi*} \nabla^* \varphi_i \nabla^* \varphi_j \right) - \sum_{i<j}^{m-1} \left(k^{\eta*} \nabla^* \eta_i \nabla^* \eta_j \right) \qquad \text{(S13)}$$

where $\tau^* = \tau \cdot f_0 M / \Delta x^2$, $L^* = L \cdot \Delta x^2 / M$, $\alpha_{ij}^* = \alpha_{ij} / f_0$, $A^* = A / f_0$, $B^* = B / f_0$, $k_{ij}^{\varphi*} = k_{ij}^{\varphi} / f_0 \Delta x^2$, $k^{\eta*} = k^{\eta} / f_0 \Delta x^2$ $\Delta^* = \Delta \cdot \Delta x^2$, $\nabla^* = \nabla \cdot \Delta x$, $\mathbf{r}^* = \mathbf{r} / \Delta x$. Here, Δx and f_0 are the length scale and

energy scale, respectively. Equation (S9) and (S10) are solved using Fourier-Spectral method with $\Delta x^* = \Delta x / \Delta x \equiv 1$ and $\Delta \tau^* = 0.2$:

$$\tilde{\varphi}_2^{n+1} = \frac{\tilde{\varphi}_2^n - g^2 \Delta \tau^* \cdot \left[\tilde{f}_{\varphi_2}^{*n} + g^2 (k_{12}^{\varphi *} + k_{13}^{\varphi *} - k_{23}^{\varphi *}) \tilde{\varphi}_3 + g^2 (k_{12}^{\varphi *} + k_{14}^{\varphi *} - k_{24}^{\varphi *}) \tilde{\varphi}_4 \right]}{1 + 2 k_{12}^{\varphi *} \Delta \tau^* \cdot g^4} \quad \text{(S14)}$$

$$\tilde{\eta}_i^{n+1} = \frac{\tilde{\eta}_i^n - L^* \Delta \tau^* \cdot \left[\tilde{f}_{\eta_i}^{*n} - k^{\eta *} g^2 \sum_{j=1}^{m} \tilde{\eta}_j^n \right]}{1 + k^{\eta *} L^* \Delta \tau^* \cdot g^2} \quad \text{(S15)}$$

$$\begin{aligned} f_{\varphi_2}^* = {} & 2 \left[\varphi_2 (\alpha_{12}^* \varphi_1^2 + \alpha_{23}^* \varphi_3^2 + \alpha_{24}^* \varphi_4^2) - \varphi_1 (\alpha_{12}^* \varphi_2^2 + \alpha_{13}^* \varphi_3^2 + \alpha_{14}^* \varphi_4^2) \right] \\ & + 2 A^* \varphi_2 g_\eta - B^* (1 - \varphi_2) \sum_{i=1}^{m} \eta_i^2 \end{aligned} \quad \text{(S16)}$$

$$f_{\eta_i}^* = A^* \varphi_2 \left(2\eta_i^3 - 2\eta_i + 3\eta_i \cdot \sum_{j \neq i} \eta_j^2 \right) + 2 B^* (1 - \varphi_2) \eta_i \quad \text{(S17)}$$

Simulation parameters. We list in Table S2 the parameters used for the dewetting simulation. The wetting angles of the metal film on the substrate and pillars, corresponding to these parameters, are: $\theta_s = \theta_p \approx 142^{\circ}$, which are within the range of reported values for the wetting angle of gold on silicon at 400°C. [2]

Gradient energy coefficients	Value
Free energy constants	
α_{ij}^*	1
A^*	1
B^*	2
$k_{12}^{\varphi*} = k_{13}^{\varphi*} = k_{14}^{\varphi*}$	0.5
$k_{23}^{\varphi*} = k_{24}^{\varphi*}$	1.6
$k^{\eta*}$	0.1
Kinetic coefficient	
L^*	100
Grain number	
m	20 for simulation of pinning effect<br>1 for others

Table S2 | Simulation parameters.

Simulation setup. Before executing the main routine of the phase field simulations, we first generate the SiO_x substrate, the HSQ nanopillars and the as-deposited metal film according to the given geometry parameters including: the period of the nanopillar array, p, the diameter of pillars, d, the height of pillars, h, and the thickness of metal film, t. It should be noted that the geometry generated here is not smooth but in a discrete manner with grid size Δx, so that we have non-dimensional parameters of: $p^* = \langle p/\Delta x \rangle$, $d^* = \langle d/\Delta x \rangle$, $h^* = \langle h/\Delta x \rangle$ and $t^* = \langle t/\Delta x \rangle$, where the $\langle \cdot \rangle$ means rounding of the corresponding values.

In comparison with our experiments, we find that the non-dimensional time is: $\tau^* \approx 22 \cdot \tau \cdot \left(p^*/p \right)^2$. We impose an initial perturbation of one grid to the surface of metal film to

consider the effect of surface roughness on the dewetting process. After the generation of initial geometry, the phase field routine will then be executed based on the model and parameters described above. As mentioned before, although our model incorporates the grain structure evolution, most of our simulations are still executed with single crystal condition in order to accelerate the calculation speed.

Analytical derivations

Critical periodicity of the Cassie-Baxter (CB) state. For simplicity, we assume that for the nanoaperture phase, the metal surface is flat and its thickness is the same as the height of the nanopillars, and for CB state [3], both the top and the bottom surfaces/interfaces are flat. Representing the diameter of the nanopillars as d , the height as h and the periodicity as p . We can obtain the surface/interface energy per unit area for the nanoaperture phase as:

$$E_{apt} = \frac{\pi dh}{p^2}\xi_{AuHSQ} + (1-\frac{\pi d^2}{4p^2})\xi_{AuSi} + \frac{\pi d^2}{4p^2}\xi_{HSQ} + (1-\frac{\pi d^2}{4p^2})\xi_{Au} \qquad \text{(S18)}$$

and the surface/interface energy per unit area for CB phase as:

$$E_{CB} = \frac{\pi dh}{p^2}\xi_{HSQ} + (1-\frac{\pi d^2}{4p^2})\xi_{Si} + (1-\frac{\pi d^2}{4p^2})\xi_{Au} + \xi_{Au} + \frac{\pi d^2}{4p^2}\cdot\xi_{AuHSQ} \qquad \text{(S19)}$$

where, ξ_{Au} , ξ_{Si} , ξ_{HSQ} , ξ_{AuSi} and ξ_{AuHSQ} are the energy densities of Au surface, Si surface, HSQ surface, interface of Au and Si, and interface of Au and HSQ, respectively. Here we assume that $\xi_{HSQ} = \xi_{Si}$ and $\xi_{AuHSQ} = \xi_{AuSi}$. So the wetting angle is:

$$\cos(\theta) = \xi_{Si}/\xi_{Au} - \xi_{AuSi}/\xi_{Au} = \xi_{HSQ}/\xi_{Au} - \xi_{AuHSQ}/\xi_{Au} \qquad \text{(S20)}$$

When the periodicity p is smaller than a critical value, the energy of nanoaperture phase will be higher than the CB phase. The critical periodicity can be derived by using $E_{apt} = E_{CB}$ and Equations of S18, S19 and S20 as:

$$p_{cri} = \sqrt{\frac{\pi d(d/2 - h)\cos(\theta)}{1 + \cos(\theta)}} \qquad \text{(S21)}$$

Critical (metal thickness – pillar height) difference that results in the Wenzel state. Besides Cassie–Baxter state, in order to obtain nanoaperture arrays with high yield, we also need to avoid the formation of Wenzel states, [4] which are wetting states where metal films form conformally over and between nanopillars. In our fabrication technique, the formation of Wenzel states can be caused by the over-deposition of metal materials (see Figure S3 for experimental observation of increasing thickness of the metal film relative to the height of the nanopillars). Thus, the thickness of the metal film with respect to the height of the nanopillars is an important parameter. The critical value of the difference between the metal film thickness and height of the nanopillars when the Wenzel state occurs, Δh_{cri}, can be defined by a simple energetic analysis.

For simplicity, we assume that the nanoapertures maintain their cylindrical shape even when the metal thickness exceeds the nanopillar height. When the difference of film thickness and pillars' height exceeds a critical value, the nanoapertures will tend to close and the Wenzel state will appear.

The surface/interface energy per unit area for the nanoaperture phase is:

$$E_{apt} = \frac{\pi dh}{p^2}\xi_{AuHSQ} + (1 - \frac{\pi d^2}{4p^2})\xi_{AuSi} + \frac{\pi d^2}{4p^2}\xi_{HSQ} + (1 - \frac{\pi d^2}{4p^2})\xi_{Au} + \frac{\pi d \Delta h}{p^2}\xi_{Au} \qquad \text{(S22)}$$

and the surface/interface energy per unit area for the Wenzel phase is:

$$E_{WZ} = \frac{\pi dh}{p^2}\xi_{AuHSQ} + (1 - \frac{\pi d^2}{4p^2})\xi_{AuSi} + \frac{\pi d^2}{4p^2}\xi_{AuHSQ} + \xi_{Au} \qquad \text{(S23)}$$

Using $E_{apt} = E_{WZ}$ and Equation S20, we have:

$$\Delta h_{cri} = \frac{d}{4} \cdot [1 - \cos(\theta)] \qquad \text{(S24)}$$

Using $d \approx 10$ nm and $\theta \approx 142^{\circ}$, we find that $\Delta h_{cri} \approx 5$ nm. In other words, the thickness of the metal deposited should not exceed ~ 5 nm above the height of the nanopillars. This result agrees well with the experimental observations in Figure S8. In addition, this suggests an additional mechanism that contributes to the lower periodicity limit for our method: As the metal caps dewet into the surrounding metal film, the film thickness increases. At small periodicities, the film thickness can increase significantly, promoting local Wenzel states and thus the failure to obtain nanoapertures at small periodicities. A simple calculation shows that at pillar height (and thus metal thickness) of 30 nm, and when the periodicity is smaller than 25 nm, the height increase induced by dewetting of metal caps will exceed 5 nm. Thus, when $p < 25$ nm, a pure Wenzel state will form at early stage and will then gradually transform to a pure CB state. When 25 nm $< p <$ 50 nm, localized Wenzel states and CB states will appear, depending on initial configuration and annealing time. This is again in line with our experimental observations and our simulation results (Figure S2B).

Supplementary Figures

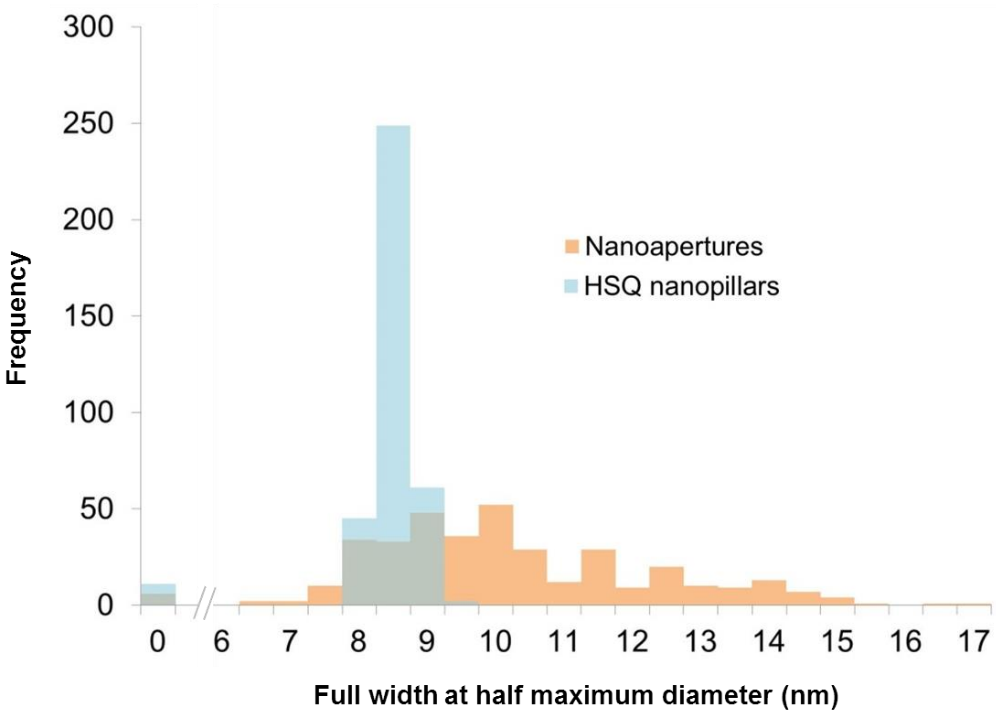

Figure S1 | Comparison between nanoaperture and initial nanopillar diameters. Histogram showing full width at half maximum diameter distribution of a typical array of nanoapertures obtained versus that of a typical array of HSQ nanopillars. The mean diameter of the nanoapertures was 10 nm and that of the nanopillars was 8 nm. Some nanopillars may collapse during development and subsequent handling. These were represented by the 0 nm bin. As can be seen, the number of missing apertures is on the order of that of the fallen nanopillars. Thus, the missing nanoapertures may be partly attributed to the imperfect nanopillar template. These reported measurements are conservative and may show a larger variation than the actual dimensions because the varying gold crystal orientations result in different local signal levels on the SEM micrograph, potentially skewing diameter measurements. This analysis was performed with a custom routine using MATLAB (Mathworks, USA).

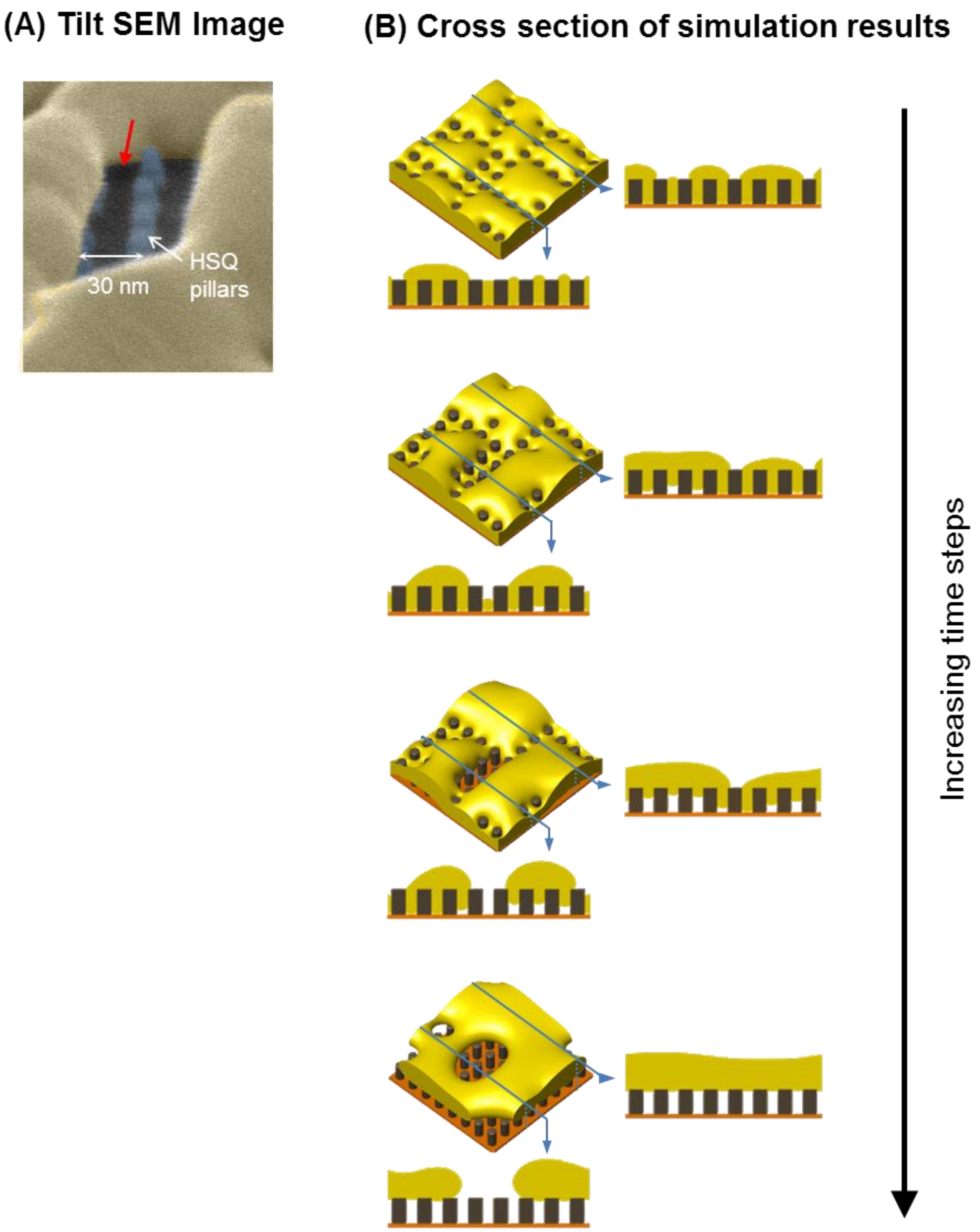

Figure S2 | Evolution from Wenzel to Cassie-Baxter state as system is driven towards equilibrium. (A) False-colored tilt SEM image of dewetted sample with h = 30nm, p = 30 nm and d ~ 10 nm. The red arrow points to gap between the gold film and the silicon substrate, indicative of a local Cassie-Baxter state. (B) Cross section view of simulation results with h = 30nm, p = 30 nm and d ~ 15 nm. Wenzel state is observed initially. With increasing simulation time steps, the gold diffuses out from between the nanopillars and away from the substrate, resulting in local Cassie-Baxter (CB) states. Eventually, pure CB states are observed. This is in line with our experimental results, although local CB states are obtained within a shorter corresponding experimental time frame. This difference can be attributed to the coalescing of the neighbouring gold caps observed experimentally, which is closer to the equilibrium CB state than the initial state in the simulations.

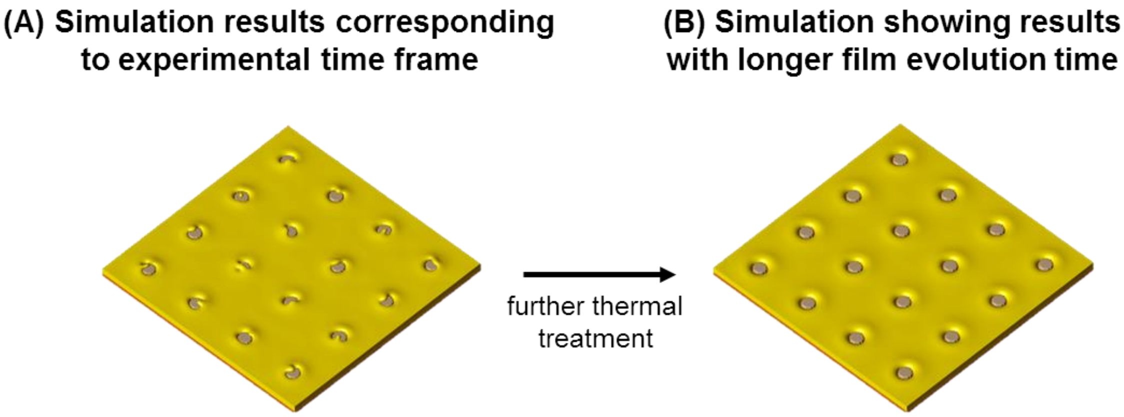

Figure S3 | Effect of thermal treatment time. Here, periodicity, p = 300 nm, and diameter, d = 70 nm. (A) Simulation results obtained with film evolution time steps corresponding to the experimental thermal treatment time frame for results in Figure 5. Similar to the experimental and simulation data in Figure 5ix, the simulation results presented here show that nanoapertures remain partially covered by gold. (B) Simulation results with longer film evolution time suggest that nanoapertures can be obtained with longer thermal treatments. However, it is also apparent here that edge retraction can occur with further thermal treatment in some template configurations. Thus, thermal treatment time is an important parameter to optimize.

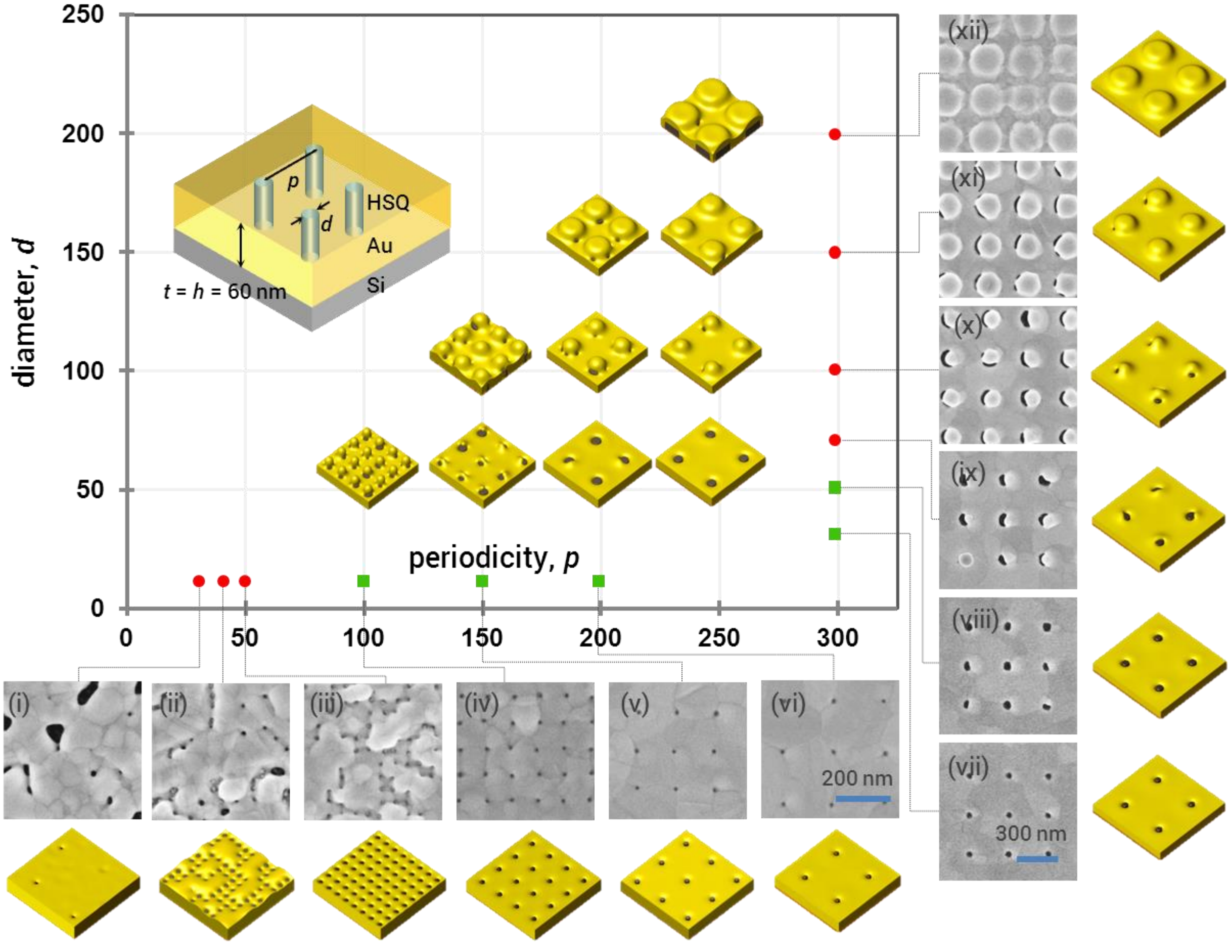

Figure S4 | Parameter variations with simulations and experiments for $t = h = 60$ nm, twice the metal thickness as that of the data set present in Figure 5 of the main text. Experimental results were obtained for (i – vi) $d \sim 10$ nm, $p = 30$ nm, 40 nm, 50 nm, 100 nm, 150 nm, and 200 nm respectively; (vii – xii) $p = 300$ nm, $d = 30$ nm, 50 nm, 70 nm, 100 nm, 150 nm, and 200 nm respectively. Simulation results were obtained for the entire parameter space, and displayed here over an area of 400 nm by 400 nm. Green squares indicate experimental parameters with which nanoaperture arrays were achieved. Red dots indicate parameters with which dewetting fails to produce aperture arrays. Nanoapertures failed to form experimentally when $p < 100$ nm, whereas simulations predict successful fabrication of nanoapertures as long as $p \geq 50$ nm. As discussed before for the case where $h = 30$ nm, due to atomic diffusion during metal deposition, the gold caps are larger than the diameters of the pillars and become adjoined with neighboring pillars. Because the deposited metal film is thicker here ($h = 60$ nm) than the set shown in the figure 5 in main text ($h = 30$ nm), the gold caps are comparatively larger, resulting in adjoining gold caps even for $p = 50$ nm (Figure S5). This suggests the importance of the initial configuration of the deposited gold film before thermal treatment.

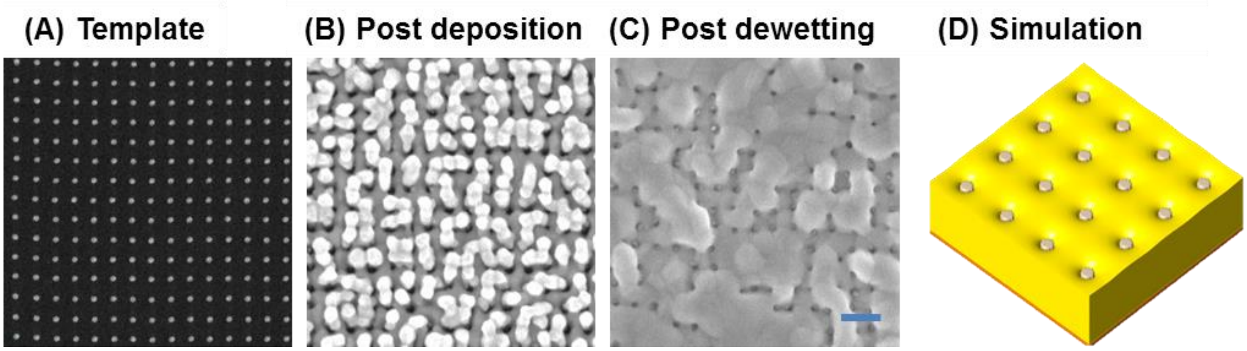

Figure S5 | Results for t = h = 60 nm, p = 50 nm Representative images of (A) template with h = 60 nm and p = 50 nm, (B) template with 60 nm of Au deposited, (C) sample after dewetting at 400°C for 10 minutes, and (D) simulation results. Simulation results indicate that dewetting should reveal aperture arrays. However, experimentally, nanoapertures were not successfully obtained as shown in (C). We note that unlike in the simulation where the gold caps were discrete, the gold caps on top of neighboring nanopillars were initially joined due to diffusion during deposition. Scale bar (A-C): 100 nm.

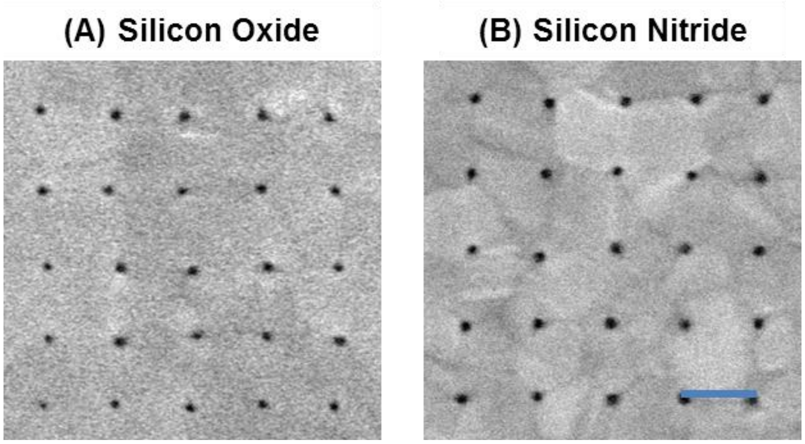

Figure S6 | HSQ nanoapertures (p = 100 nm; t=h= 55 nm) obtained with templated dewetting on (A) 100 nm thick thermally grown silicon oxide, and (B) 50 nm plasma-enhanced chemical vapor deposited silicon nitride, respectively. Scale bar: 100 nm.

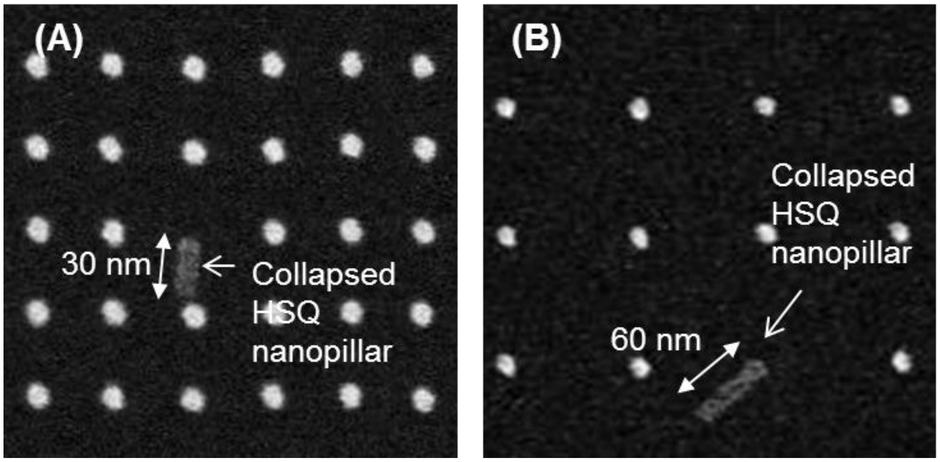

Figure S7 | To elucidate the height of the nanopillars, fallen nanopillars were imaged using the SEM. The SEM images revealed that the heights of the nanopillars were 30 nm (A) and 60 nm (B) respectively.

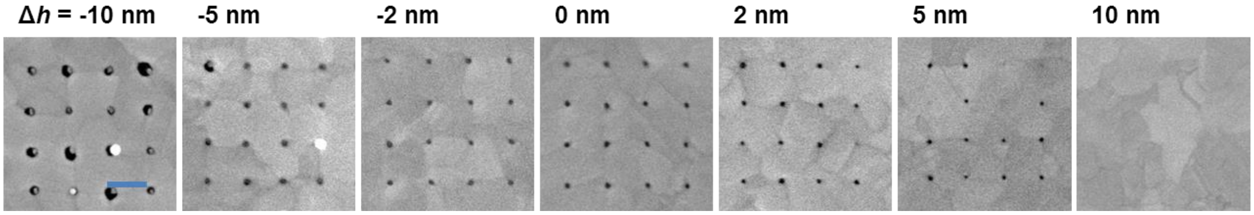

Figure S8 | Effect of metal thickness. SEM images of patterned regions with 30 nm tall nanopillars after thermal treatment for different metal thicknesses. Δh is defined as the difference between the height of the nanopillars, h, and the thickness of the deposited metal, t, i.e. $\Delta h = t - h$. Scale bar: 100 nm.

Supplementary Video

Supplementary Video M1 | Simulated film evolution. Upon heating, the gold caps deposited on top of the nanopillars merge into the surrounding gold film via thermally-induced accelerated diffusion. Over time, the gold caps diffuse completely into the surrounding gold film, revealing the nanoapertures.

References

1. Jiang, W., Bao, W., Thompson, C. V. & Srolovitz, D. J. Phase field approach for simulating solid-state dewetting problems. *Acta Mater.* **60,** 5578–5592 (2012).

2. Ressel, B., Prince, K. C., Heun, S. & Homma, Y. Wetting of Si surfaces by Au–Si liquid alloys. *J. Appl. Phys.* **93,** 3886 (2003).

3. Cassie, A. B. D. & Baxter, S. Wettability of porous surfaces. *Trans. Faraday Soc.* **40,** 546–551 (1944).

4. Wenzel, R. N. Resistance of solid surfaces to wetting by water. *Ind. Eng. Chem.* **28,** 988–994 (1936).
